# Analysing Digital Engagement Patterns: A Machine Learning Investigation into Social Anxiety Among Adolescents with ADHD

**DOI:** 10.3390/jcm13237461

**Published:** 2024-12-07

**Authors:** Roxana Șipoș, Tudor Văidean, Andreea-Marlena Răpciuc, Costina-Ruxandra Poetar, Elena Predescu

**Affiliations:** 1 Department of Neuroscience, Psychiatry and Pediatric Psychiatry, “Iuliu Hatieganu” University of Medicine and Pharmacy, 57 Republicii Street, 400489 Cluj-Napoca, Romania; 2 Department of Clinical Psychology and Psychotherapy, Faculty of Psychology and Educational Sciences, Babeş-Bolyai University, 37 Republicii Street, 400015 Cluj-Napoca, Romania; 3 Clinical Department of Nephrology, County Emergency Clinical Hospital Cluj, 3-5 Clinicilor Street, 400006 Cluj-Napoca, Romania

**Keywords:** screen time, adolescents, ADHD, anxiety symptoms, digital engagement

## Abstract

**Background:** The relationship between screen time and mental health among adolescents, particularly those identified as “digital natives”, has become increasingly pertinent, especially in the context of heightened digital engagement during the COVID-19 pandemic. This study aims to examine the associations between digital engagement patterns, ADHD severity, and anxiety symptoms in adolescents aged 11–18. **Methods:** A cross-sectional observational study was conducted with a sample of 97 adolescents diagnosed with ADHD. Participants completed validated questionnaires assessing daily digital activities and anxiety symptoms while caregivers provided additional contextual information. Data were analyzed using Random Forest regression to identify relationships between screen time, ADHD severity, and anxiety symptoms. **Results:** The findings indicate an average screen time of 6.6 h on weekdays and 8.1 h on weekends, with social media emerging as the primary activity, particularly among female participants. Notable gender differences were identified, with family dynamics influencing digital engagement; family disputes had a more significant impact on girls than boys. Furthermore, specific anxiety traits, i.e., physical symptoms and harm avoidance, significantly predicted time spent on social media across all genders considered in the study, while others, namely social anxiety and separation anxiety, were less valuable. **Conclusions:** This study highlights the complex interplay between screen time, ADHD, and anxiety symptoms in adolescents. These findings underscore the importance of tailored interventions that address the nuanced relationships between digital engagement and mental health in this population, warranting further research to develop effective management strategies.

## 1. Introduction

Over the past two decades, the relationship between screen time and mental health has been a subject of intense debate. This issue is particularly significant for the generations born after 2000, often referred to as “digital natives” [1]. These individuals, who have grown up in a digital environment, exhibit fundamentally different psychosocial dynamics and interpersonal relationships compared to previous generations [2]. Despite extensive research, the effects of this digital transformation remain incompletely understood. Consequently, the World Health Organization (WHO) and the American Academy of Pediatrics (AAP) have issued guidelines recommending no screen exposure for children under 2 years old and limiting it to one hour per day for children aged 2 to 5 [3,4]. Contrarily, studies suggest that screen exposure begins as early as four months of age [5], and screen use has increased dramatically, becoming a ubiquitous part of daily life [6]. A meta-analysis in 2022 revealed that 75% of children under 2 and 64% of children aged 2 to 5 exceed the recommended screen time limits [7].

For older children and adolescents, no clear guidelines exist despite their heightened susceptibility to digital technologies [8]. The COVID-19 pandemic exacerbated this issue, increasing adolescents’ screen time from 2.7 to 4.1 h per day [9], with nearly 60% of adolescents exceeding 10 h of screen time during peak pandemic periods [10]. Studies have also documented a rise in psychiatric complaints, particularly anxiety and depression, associated with increased screen time during the pandemic [11].

Extensive research has aimed to identify the consequences of prolonged screen exposure among the pediatric population. One primary concern is the impairment of attentional capacity, even with brief exposures [12]. Other reported effects include emotional dysregulation, impulsivity, and difficulties in peer relationships [13,14]. Furthermore, prolonged screen time has been linked to internalizing and externalizing symptomatology, with internalizing issues attributed to indirect effects such as social withdrawal and sleep disturbances, while externalizing issues are correlated with content type [7]. However, findings are often contradictory, with some studies and meta-analyses reporting no adverse effects or even positive impacts of certain digital activities on mental health [15,16]. For instance, moderate screen time (30 min to 2 h per day) has been associated with better psychological well-being compared to no screen time [17].

The differentiation of digital activities is crucial, as their distinct effects on mental health have been recognized by several systematic reviews [8,18,19,20]. Adolescents typically spend most screen time watching television, playing video games, and using social networks [21]. Studies examining these activities have yielded mixed results. For instance, television watching has been linked to attentional impairments [22] and anxiety disorders [23]; although some studies suggest anxiety reduction is associated with this activity [24]. Similarly, video game use has been identified as a predictor of anxiety and depressive symptoms [25]; though other studies do not confirm these findings [21,26]. Social networks are particularly contentious, with some research indicating links to increased anxiety, depression, and loneliness [27,28,29], while others highlight potential benefits in specific contexts, such as self-identity expression leading to well-being [30].

The strong impact of social networks on mental health is partly due to the objective nature of virtual rejections (likes, comments, etc.), algorithmic amplification of content, and the propagation of unrealistic standards [12,14]. Anxiety, including social anxiety, appears to be a common correlate of digital activities among adolescents. However, this relationship may be bidirectional, with anxious adolescents using digital media as an escape from unsatisfactory realities [8,31]. Reducing screen time has been shown to improve internalizing symptoms in some studies [32,33].

Given the aspects outlined above, the issue of prolonged screen time has broader implications for adolescents with ADHD, as these consequences add to pre-existing attentional deficits, impulsivity, and social integration difficulties [34]. Up to 80% of these adolescents are excluded from social environments due to their symptoms [35,36]. This stigmatization often leads to guilt for academic and social failures, creating a cycle of social withdrawal and anxiety disorders [37]. A systematic review found that up to 50% of ADHD patients meet the criteria for at least one anxiety disorder [38], with social phobia comorbidity reaching up to 72.3% [39].

Adolescents with ADHD may turn to digital media, especially social networks, to form friendships [40]. However, the “social compensation” phenomenon, where individuals with social difficulties use electronic devices excessively, further impairs emotional and social functioning [41]. Another problematic aspect is the “digital divide” phenomenon, wherein individuals with adequate offline interaction skills tend to benefit from virtual media use, including the internet. In contrast, those who face social difficulties in real life, often observed in individuals with ADHD, are more likely to be adversely affected by electronic device use [12].

Adolescents with ADHD use electronic devices more frequently than their non-ADHD peers, averaging 5.6 h per day [42]. This behavior may be due to cognitive-behavioral mechanisms and genetic and neuroanatomical predispositions linking ADHD with prolonged screen time [43]. Video gaming is particularly appealing due to its stimulating, episodic nature, which aligns with ADHD-specific impulsivity [44]. Despite the bidirectional relationship between screen time and ADHD symptomatology, the negative effects of digital activities are more pronounced in ADHD patients compared to the general population [44,45].

Given the limited research with clear methodology and the lack of studies on adolescents with ADHD, further investigation is warranted. This study aims to explore the relationships between social anxiety, ADHD symptom severity, and screen time for various digital activities among adolescents with ADHD. We also seek to identify preferred electronic devices and digital activities and their correlation with psychopathological manifestations.

## 2. Materials and Methods

### 2.1. Study Design and Participants

We conducted a cross-sectional observational clinical study at the Pediatric Psychiatry Clinic Cluj-Napoca from October 2023 to January 2024. Participants were patients who presented with a legal representative (parent, guardian, foster parent, etc.) for initial evaluation or follow-up during this period. All adolescents and parents underwent an unstructured interview with one of the two senior child and adolescent psychiatrists involved in the study, which included the identification of potential problems followed by the targeted exploration of psychopathological manifestations as described in the form of DSM-5 criteria. In addition, the psychometric instruments described later were administered and subsequently analyzed to identify potential problems missed during the free interview.

### 2.2. Inclusion and Exclusion Criteria

To establish eligibility for study participation, the inclusion criteria were age between 11 and 18 years, a diagnosis of ADHD established according to the DSM-5 criteria, and written consent from both the caregiver and the adolescent. Exclusion criteria included cognitive impairment (IQ < 70), psychotic disorders, and deficient lexical-graphic skills that hindered the proper understanding of questionnaire items. Participants were also excluded if only one party (caregiver or adolescent) completed the necessary questionnaires, if responses were incomplete, or if the reported screen time exceeded 24 h per day in total.

### 2.3. Data Collection

Adolescents were provided with a comprehensive self-report questionnaire capturing various aspects of their daily digital and non-digital activities. Adapted from Khouja’s 2019 study, this questionnaire included sections on digital activities, preferred electronic devices, physical activity, screen time regulation, sleep patterns, and family and social factors [46]. It assessed the time allocated to watching TV programs, using streaming platforms, YouTube, video games, and social media, as well as the usage of phones, tablets, laptops, PCs, TVs, and gaming consoles. It also gathered information on physical activities, caregiver-imposed limits on screen time, sleep duration, family disputes, family aggression, school bullying, and digital bullying.

To further assess pediatric anxiety, adolescents completed the Multidimensional Anxiety Scale for Children (MASC), a 39-item self-report instrument. The MASC evaluates anxiety across four subscales: Physical Symptoms, Social Anxiety, Harm Avoidance, and Separation Anxiety [47]. Each item is scored from 0 (never true) to 3 (often true), allowing for a detailed assessment of anxiety levels in children and adolescents. This scale was previously used with Romanian samples [48].

Caregivers completed a condensed version of the screen time assessment questionnaire, corroborating the adolescents’ reports. This version focused on the same key areas: digital activities, preferred devices, physical activity, screen time limits, sleep patterns, and family/social factors. Taking into account parents’ reports also provides an additional perspective on the issues of interest, which is important in the context that reports may be influenced by communication style as well, and rigorous psychometric validation is crucial for trustworthy self-report data [49]. In cases where adolescents reported a surprisingly high involvement in digital activities (more than 12 h per day), the investigators tried to prevent the inclusion of untruthful data that could bias the results by asking follow-up questions investigating the exact structure of a typical day in the adolescent’s life, and by comparing the reports provided by adolescents with those provided by their parents in order to exclude those cases characterized by great discrepancies (difference > 2 h). Additionally, caregivers completed the ADHD Rating Scale IV (ADHD-RS IV) [50], which includes an Inattention Subscale and a Hyperactivity/Impulsivity Subscale. Each subscale consists of 9 items assessing symptoms of inattention, hyperactivity, and impulsivity, rated on a scale from 0 (never or rarely) to 3 (very often). This format allows for a comprehensive evaluation of ADHD symptoms and has demonstrated strong psychometric properties in the Romanian pediatric population [50,51].

### 2.4. Statistical Analysis

Data were recorded into a database and analyzed using R version 4.3.3 for MacOS and R Studio version 2023.12.1+402. The primary analytical method employed was Random Forest regression, executed using the ranger package. This method was selected for its ability to handle complex, non-linear relationships and interactions between variables, making it particularly suitable for our multifaceted dataset, and also its capacity to prevent overfitting. Several studies in the literature, although on other subjects, have compared different regression techniques, and in many of these, Random Forest techniques were the most accurate [52,53].

Separate Random Forest regression models were constructed for each digital activity, distinguishing between weekdays and weekends/holidays. Predictor variables included demographic, clinical, and behavioral factors: sex, age, raw scores for the ADHD-RS subscales (Inattention and Hyperactivity/Impulsivity), raw scores for the MASC subscales (Physical Symptoms, Social Anxiety, Harm Avoidance, and Separation Anxiety), and time allocated to various non-digital activities (such as outdoor activities, social interactions, and schoolwork). Additionally, several dichotomous variables were incorporated to account for binary conditions such as the administration of ADHD medication, comorbidity with depression, participation in sports, family disputes, family aggression, school bullying, and digital bullying.

Each Random Forest regression model comprised 2000 Decision Trees, a sufficiently large number to enhance the model’s stability and predictive power. Initially, we designed a training model for each outcome prediction, setting the mtry parameter, which specifies the number of variables to be sampled at each split in the tree, to 6 based on established conventions. Subsequently, we performed a hyperparameter grid search to evaluate the top 10 predictive models in terms of mtry, min.node.size, and sample.fraction. For example, if 5 of these models had an mtry value of 3, we selected mtry 3; similarly, if the majority had a sample.fraction of 0.50, we chose that value. This process allowed us to determine the final set of optimal hyperparameters for our models.

To assess the importance of each predictor variable, permutation importance was utilized. This method involves shuffling the values of each predictor variable and measuring the consequent change in the model’s performance. Unlike impurity-based importance measures, permutation importance provides a more clinically relevant indication of a variable’s significance by directly reflecting its impact on model accuracy [54]. Detailed descriptions of the hyperparameters and their optimization process for each regression model are provided in Supplementary Tables S1 and S2.

### 2.5. Ethical Considerations

This study was conducted in accordance with the Declaration of Helsinki and was approved by the Ethics Committee of Babeș-Bolyai University (approval no. 3772/8 April 2022). Written informed consent was obtained from all participants and their legal guardians. To ensure data protection and privacy, all personal information was anonymized and securely stored. Only authorized personnel had access to the data, and all analyses were conducted using de-identified data to maintain confidentiality.

## 3. Results

### 3.1. Sample Description

Out of the 143 pairs of questionnaires administered, 28 adolescent-caregiver dyads were excluded for the following reasons: 14 were completed only by the adolescent, 11 were completed only by the caregiver, and 3 were completed inadequately by both parties. This resulted in a total of 105 pairs of data initially collected. Upon further analysis, 8 pairs did not meet the inclusion criteria, as the total screen time allocated to various activities exceeded 24 h per day. Consequently, 97 pairs of responses were subjected to statistical analyses, with the majority completed online (n = 63).

Among the 97 adolescents, the majority (n = 52) were female. The age distribution of the participants was not normally distributed across the entire sample (Shapiro–Wilk *p* = 0.001), with a median age of 14.5 (±2.074) years. Significant age differences were found between sexes (*p* = 0.001); the mean age among boys was 13.807 (±2.169) years, while among girls, it was 15.137 (±1.790) years, with normal distributions within each sex group. Depressive disorders were the most frequent comorbidity associated with ADHD, diagnosed in 44.32% of the study participants. The exact distribution of diagnoses is presented in Table 1.

At the time of inclusion in the study, 25 participants were receiving specific ADHD treatment, with 18 of them undergoing treatment with Methylphenidate and 7 with Atomoxetine. Additionally, 66 participants were on other psychotropic medications.

Self-reported data indicated that spending time with friends was the preferred non-digital activity among female participants, both during the week and on weekends and vacations. In contrast, boys reported allocating most of their time to homework during the week and outdoor activities during weekends and vacations. These self-reports were largely consistent with the reports provided by parents, as shown in Table 2.

Regarding digital activities, the adolescents in this study reported an average daily screen time of approximately 6.636 h on weekdays and 8.146 h on weekends and holidays, with individual usage ranging from 30 min to 19.2 h per day. Statistically significant differences determined using the Kruskal–Wallis test (due to unequal variances suggested by Levene’s test) were observed between genders in terms of social media use and video gaming, both during the week and on weekends and holidays. The mean values reported by adolescents, along with those provided by parents, are presented in Table 3.

When asked about their preferred device, most participants (n = 66) indicated the smartphone, with an average usage of 5.144 (±3.135) hours per day across the entire sample. Detailed results for other variables included in the screen time assessment questionnaire are presented in Table 4. Additionally, the scores from the MASC and ADHD-RS instruments are summarized in Table 5.

In addition to these factors, adolescents were queried about their primary sources for staying informed about current events. The results indicated that girls predominantly rely on social media as their main source of information (n = 23), followed by television (n = 11). In contrast, boys exhibited a different pattern, with the majority preferring news broadcasts on television (n = 18), followed by news disseminated through social media (n = 11).

### 3.2. Prediction of Time Allocated to Different Types of Digital Activities

#### 3.2.1. Social Networks

The regression model explained 33.562% of the variance in the mean weekly time that adolescents with ADHD allocate to social networks, with a root mean square error (RMSE) of 2.360. Permutation importance analysis identified the most significant predictive variables, ranked as follows: sex (0.918), age (0.898), and ADHD medication (0.798). Given the substantial influence of gender as a predictor, a differentiated approach based on sex was deemed more appropriate than a generalized model for all adolescents. This distinction enhances our understanding of social media usage patterns within this population.

**Figure 1:**
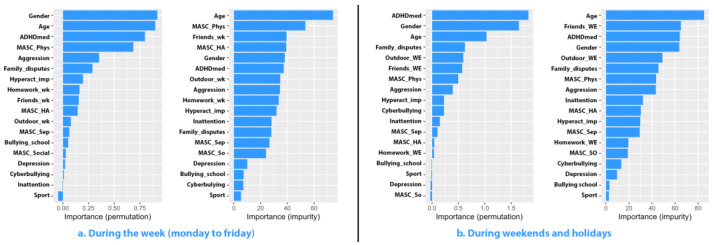
Importance of predictors for social media usage among adolescents of both genders.

For weekends and holidays, the predictive power of the model was 42.980%, with a root mean square error (RMSE) of 2.401. The most significant predictors of the variable of interest were, in order, ADHD medication (1.827), sex (1.648), and age of the participants (1.030). Notably, age emerged as the primary factor in the regression model’s decision tree construction, indicated by an impurity score of 85.093. Consistent with previous analyses, we employed differentiated models based on the gender of the adolescents to enhance predictive accuracy. The importance of all variables is presented in Figure 1.

##### Social Networks Among Female Participants

In the gender-differentiated analysis for female participants, the model accurately predicted social media usage during the week with an accuracy of 21.062% (RMSE: 2.893). The most predictive factors identified were family disputes (1.269) and intrafamilial aggression (1.069), followed by the age of the participants (1.064). Notably, these variables ranked among the top four considered in the construction of the model’s decision trees based on their associated Mean Decrease in Impurity (MDI). The average time allocated to social media was significantly higher in the presence of family disputes, with a mean of 5.909 ± 3.734 h per day compared to 2.625 ± 1.959 h per day (*p* < 0.001). Similarly, in cases of intrafamilial aggression, the average usage increased to 8.750 ± 5.017 h per day versus 3.397 ± 2.418 h per day (*p* < 0.001). A positive and significant correlation was observed with age (*p* = 0.003; Pearson’s r = 0.400).

Among the symptoms of ADHD, hyperactivity/impulsivity was found to be significant, with a predictive importance score of 0.386, ranking fifth overall. In contrast, inattention showed no predictive value, with a score of −0.077. Physical manifestations associated with anxiety had a predictive importance of 0.433, placing fourth in the hierarchy, while avoidance behaviors contributed to a lesser extent, with a score of 0.077. Notably, separation anxiety (−0.054) and social anxiety (−0.139) exhibited negative predictive values.

**Figure 2:**
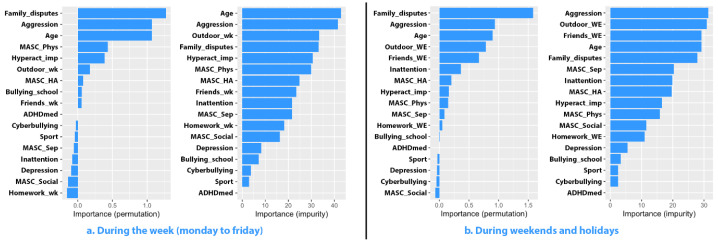
Importance of predictors for social media usage among female participants.

Despite these differences, all four facets of anxiety contributed to the overall purity of the regression model, as illustrated in Figure 2. This indicates the comprehensive influence of anxiety dimensions on the predictive capacity of the model.

Social media usage during weekends and holidays was predicted with an accuracy of 29.817%, yielding a root mean square error (RMSE) of 2.902. The most significant predictive factors identified were intrafamilial disputes (1.5799), followed by intrafamilial aggression (0.937) and age (0.899). The predictive value of these variables was evident in the substantial increase in social media time allocated in the presence of family disputes, with participants spending an average of 6.750 ± 3.924 h per day compared to 3.250 ± 2.145 h per day (*p* < 0.001). Similarly, in cases of aggression, the average usage was 9.750 ± 4.855 h per day versus 4.076 ± 2.681 h per day (*p* < 0.001). A significant positive correlation with age was also observed (*p* = 0.003; Pearson’s r = 0.403).

Additionally, time spent outdoors (0.789) and activities with friends (0.665) demonstrated high predictive importance. Post-hoc statistical analyses revealed significant positive correlations between these activities and social media usage, with outdoor activities showing a correlation of *p* = 0.003 (Pearson’s r = 0.406) and activities with friends also at *p* = 0.003 (Pearson’s r = 0.409).

Unlike previous predictions, inattention contributed more significantly than hyperactivity/impulsivity in terms of the model’s predictive power (permutation: 0.366 vs. 0.162) and purity (19.914 vs. 16.480). Among the facets of anxiety, social phobia was the only one without predictive value (−0.069), ranking last in the hierarchy of all variables included in the model.

##### Social Networks Among Male Participants

For male participants, the model predicted social media usage during the week with lower accuracy, R^2^ = 0.1681 and RMSE = 1.3116. The most significant predictors were time allocated to homework (0.270), digital bullying (0.212), age (0.199), and symptoms of separation anxiety (0.189). Post-hoc analyses revealed a significant negative correlation between the dependent variable and time allocated to homework (*p* = 0.001, Pearson’s r = −0.471). Additionally, a significant positive correlation was found with age (*p* = 0.001, Pearson’s r = 0.470), while a weak negative correlation was observed with separation anxiety (*p* = 0.018, Pearson’s r = −0.352). Furthermore, screen time for this activity was significantly higher in the group subjected to cyberbullying, with an average of 3.200 ± 1.643 h per day compared to 1.100 ± 1.247 h per day (*p* = 0.001). ADHD medication status (0.080), time spent with friends (0.047), and hyperactivity (0.012) also contributed to the predictive model. Boys receiving ADHD treatment spent significantly less time on social media compared to untreated peers (0.867 ± 0.719 vs. 1.567 ± 1.649 h/day), although this difference was not statistically significant (*p* = 0.125).

**Figure 3:**
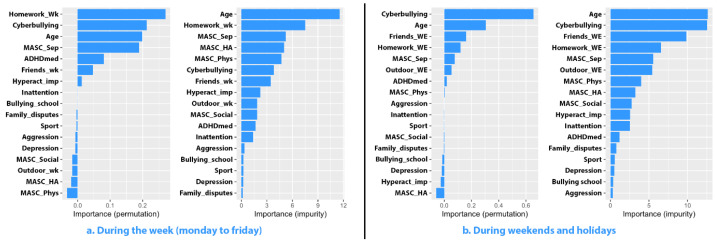
Importance of predictors for social media usage among male participants.

Separation anxiety manifestations (impurity: 5.221) ranked third in predictive importance, following age and time allocated to homework, while avoidance behaviors (5.008) and somatic complaints associated with anxiety (4.700) were fourth and fifth, respectively. In contrast, social anxiety exhibited lower predictive importance (1.834), ranking tenth overall. Boys allocated significantly more time to social media during weekends and holidays, with the model predicting this usage with an accuracy of 27.271% (RMSE = 1.4377). Cyberbullying emerged as the primary predictive factor (permutation: 0.655) and the second most significant contributor to model purity (impurity: 12.528), closely following age (12.621). Specifically, boys with ADHD who were victims of cyberbullying spent 3.3 times more time on social media compared to their peers who did not experience such incidents (4.300 ± 1.304 vs. 1.282 ± 1.412, *p* < 0.001). The contribution of other factors, such as time allocated to non-digital activities, including those spent with friends (permutation: 0.160), homework (0.120), outdoor activities (0.052), or symptoms of separation anxiety (0.078), showed much lower predictive value. Meanwhile, ADHD characteristics and other facets of anxiety were irrelevant in terms of prediction. The results obtained are graphically summarized in Figure 3.

#### 3.2.2. Video Games

The use of video games during the week was predicted with low accuracy, at 6.96% (RMSE = 1.125). The most important predictor in this case was the participants’ sex (0.773), followed at a considerable distance by age (0.114), somatic symptoms associated with anxiety (0.087), and anxious avoidance (0.036). Regarding the time dedicated to this activity during weekends and holidays, the predictive accuracy of the model was higher (R^2^ = 0.111, RMSE = 1.3693). Again, sex was the primary predictor (0.761). Given these results, separate predictive models for girls and boys were developed.

**Figure 4:**
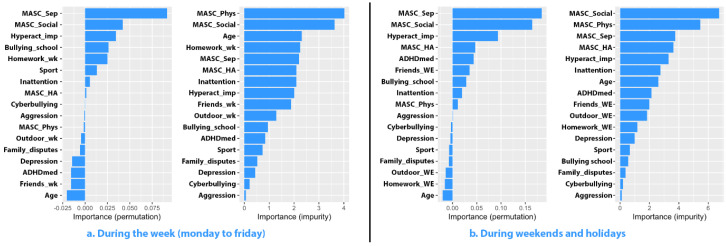
Importance of predictors for video game use among girls with ADHD.

The accuracy of the model was low in predicting the time allocated by girls with ADHD for video games during the week (R^2^ = 0.0560, RMSE = 0.8070). Separation anxiety exhibited the highest permutation importance (0.091), followed by social anxiety (0.042), hyperactivity (0.034), and school bullying (0.025). Post-hoc analyses revealed a positive, albeit nonsignificant, correlation between the dependent variable and the two facets of anxiety (with separation anxiety: *p* = 0.285, Pearson’s r = 0.151; with social phobia: *p* = 0.166, Pearson’s r = 0.195). Although a similar weight of permutation importance for the variables was obtained in the predictive model for the time allocated to this activity during weekends and holidays, it had a very low predictive capacity of only 1.27% (RMSE = 1.2091). The graphs corresponding to the two models are presented in Figure 4. The model used to predict the time that boys with ADHD allocate to video games was ineffective.

#### 3.2.3. Streaming Platforms

The predictive accuracy of the model for the time allocated to streaming platforms from Monday to Friday was null (R^2^ = −0.004), while the model for predicting usage during weekends and holidays had a predictive power of 7.027% (RMSE = 1.3079). The most significant predictive factors were time spent with friends (0.175), separation anxiety (0.119), and symptoms of social phobia (0.059). Among ADHD symptoms, inattention had a minor contribution to this model (0.035), while hyperactivity had minimal influence on predictions (0.001).

#### 3.2.4. YouTube and Similar Platforms

The predictive model for this type of digital activity performed poorly for both weekday usage (R^2^ = 0.0363, RMSE = 1.4644) and weekend/holiday usage (negative R^2^, indicating model inefficiency due to mathematical aberration).

#### 3.2.5. Watching TV

In this case, the constructed regression model was also aberrant, as indicated by the negative R^2^ obtained for both TV watching during the week and the time allocated to this activity on weekends and holidays.

## 4. Discussion

This study aimed to elucidate the relationship between digital activities and the psychological functioning of adolescents with ADHD, employing a methodology that conceptualizes screen time as an emergent phenomenon of the brain–behavior–environment covariation [55]. One of the primary findings from both our results and the analysis of existing empirical data is the presence of distinct gender-specific trends in the use of various digital media.

Our findings reveal that social media is the predominant digital activity for girls with ADHD, constituting more than half of their total screen time. Conversely, boys with ADHD exhibit a more balanced distribution of screen time across activities such as YouTube, video games, and social media. These gender-specific trends are consistent with findings from other studies on adolescent populations [56,57,58]. Adolescence, characterized by hormonal and neurodevelopmental changes, is a critical period of social reorientation, where there is a decreased focus on family and an increased focus on peers, with heightened sensitivity to social rewards processed with an emphasis on emotional aspects [59].

The data suggest that girls value social interactions more than boys, developing closer relationships that they perceive as central to their self-identity [60,61]. This emphasis on social interactions can have negative effects, such as a higher frequency of conflicts with friends and an increased rate of suicide attempts resulting from social isolation among girls [62,63]. Over the last decade, adolescents have increasingly socialized in digital environments, with this trend being particularly pronounced among girls with ADHD compared to both girls without ADHD and boys [64,65].

Literature suggests that social media use may have a stronger psychological impact on girls than boys, with potentially detrimental effects [57,66,67]. To better understand this impact, it is useful to decompose social media use into its components, as different aspects of social media may differently affect adolescents’ well-being. Self-presentation, the motivation to portray one’s identity online to achieve a digital social status measurable through likes and followers, is strongly associated with internalization problems among girls [66]. Neuroimaging studies support the importance of digital status indicators on mental health, showing increased neural activation in regions involved in reward processing, social cognition, imitation, and attention when adolescents viewed highly rated posts [68]. In summary, girls with ADHD demonstrate a higher engagement with social media, reflecting greater interest and responsiveness to digital self-presentation. In contrast, boys tend to allocate their time more evenly across various digital platforms, indicating a more balanced approach to their online activities.

### 4.1. Social Media Use in Girls

Our study reveals that social media usage among adolescents with ADHD is predominantly predicted by age, regardless of gender. This finding aligns with existing literature indicating a gradual increase in digital media use during adolescence, which peaks around age 20 and then plateaus into adulthood, where motivations such as information seeking and boredom reduction become more prominent [69]. These data suggest that social media use not only fulfills a desire to connect with peers but may also reflect temporal variations in parenting practices and adolescents’ desire to conform to peer groups.

Gender-specific trends further emerged, with intrafamilial disputes significantly predicting social media use among adolescent girls with ADHD, a factor that appears irrelevant for boys. Girls tend to be more reactive to interpersonal conflicts [70], seeking social support through social media as a coping mechanism [59]. In contrast, boys are more likely to develop externalizing problems in response to such events [71]. This positive correlation between parental disputes and problematic social media use is supported by other studies, suggesting that adolescents might use social media to regain self-esteem affected by family disputes or due to beliefs that digital interactions are safer than physical ones in conflict-ridden families [72]. Moreover, the frequent occurrence of family conflicts in households with ADHD children [73,74], reported at 35.05% for disputes and 11.34% for aggression in our study, underscores their prevalence, with no significant gender differences in exposure. These values, although high, are not surprising, as there are numerous studies in the literature demonstrating elevated rates of conflict, hostility, and corporal punishment in families with children with ADHD [75,76,77]. The explanation for this phenomenon is most likely a bidirectional one, with evidence supporting, on the one hand, the impact that child psychopathology has on parental well-being and burden [78] and, on the other hand, the impact of parental stress on children’s mental health [79].

Anxiety also influences the amount of time girls with ADHD spend on social media. Our results indicate that somatic manifestations of anxiety, such as tremors, dizziness, chest pains, dissociative symptoms, changes in heart rate, and nausea, partially predict social media use. During the COVID-19 lockdown, social media was often used as a coping mechanism for such symptoms [80]. However, social media also facilitated the spread of negative emotions, or “social contagion”, which adolescents are particularly vulnerable to [81,82]. Higher engagement in positive upward comparisons, common among those who ruminate, is associated with experiencing more physical symptoms [83], suggesting a bidirectional relationship where anxiety can be both a cause and a result of prolonged digital exposure [84].

Harm avoidance also emerged as a predictive facet of anxiety, corroborated by literature showing a positive correlation between this trait and problematic social media use, with stress levels mediating the effect [85]. Anxious avoidance might be a risk factor for problematic social media use, further mediating the relationship between avoidance tendencies and low self-esteem [86]. This trait is more predictive of social media use during weekends and holidays than during the week, possibly because social media use is more normative during school days and fills limited free time [87]. Its prolonged use during weekends, despite other available activities, indicates pronounced avoidance traits.

Additionally, separation anxiety predicts social media use to a small extent, particularly during weekends and holidays. Although there is limited literature on this relationship, one study associates separation anxiety, more frequent in girls, with Instagram use [88]. Girls with such traits may prefer social media as it allows them to relax while staying connected to their families.

Contrary to our expectations, social anxiety does not seem relevant to social media consumption in this population. While some studies on emerging adults suggest a positive correlation [89], other research indicates that social media might reduce social phobia symptoms, but only when used actively to develop communication skills [60]. Social media may thus benefit those with social anxiety by compensating for real-world support deficits and offering opportunities to overcome communication challenges [90].

### 4.2. Social Media Use Among Boys

Among boys with ADHD, the amount of time spent on homework during weekdays emerged as the primary non-digital activity and the most significant predictor of social media use, showing a moderate negative correlation. More time dedicated to homework logically results in less free time for social media and other activities. This relationship diminishes during weekends and holidays when homework demands are lower. Literature indicates that homework time might indirectly reflect ADHD severity, though the direction of this relationship remains unclear. Some studies suggest that boys with less severe ADHD might focus better on tasks, while others propose that those with more severe ADHD need more time to complete assignments [85,86]. However, post-hoc analyses in our study found no significant correlation between homework time and ADHD scores, suggesting that the most likely explanation is simply less available time for social media during weekdays.

Cyberbullying also significantly predicted extended social media use during weekends and holidays and was the second most relevant factor for social media use during school days among boys with ADHD. Although girls tend to experience cyberbullying more frequently, it does not predict their social media use. This discrepancy likely reflects different coping mechanisms: boys may become more aggressive and spend more time on social media to continue conflicts or seek new friends [91], whereas girls typically seek social support from parents or peers [92]. This difference is also supported by higher rates of non-suicidal self-injury among girls [93].

A notable gender difference is the relationship between social media use and separation anxiety. For boys, separation anxiety significantly predicts social media use during school days but not during weekends and holidays. This negative correlation suggests that boys may seek parental comfort rather than turning to social media as a refuge. Other anxiety components, such as harm avoidance and somatic manifestations relevant for girls, do not have predictive value for boys.

Additional factors, including age, time spent with friends and outdoors, and ADHD medication, also contribute to predicting social media use among boys. Notably, ADHD medication use during school days is associated with less time spent on social media, even though the severity of ADHD symptoms does not predict social media use. Literature cautiously suggests that medications like methylphenidate might help treat internet addiction [94]. This hypothesis is supported by the shared genetic and neurostructural basis of ADHD and prolonged social media use [43], as well as the common developmental intertwining of ADHD and addictive behaviors [55].

In contrast to girls, intrafamilial disputes do not predict social media use among boys, indicating different coping mechanisms. This is supported by literature describing a transactional model between such conflicts and behavioral disorders in boys [95].

### 4.3. Video Game Use Among Girls

The prediction of video game use among girls with ADHD proved challenging, achieving only a 5.6% predictive accuracy. This suggests that, unlike social media, video gaming is a more recreational and varied activity for adolescents, driven by a range of intrinsic motivations such as the desire for autonomy, competence, and relatedness [96,97]. As our study did not assess such factors, the results obtained do not allow us to draw clear conclusions of practical value. However, there are some aspects that should be taken into account by further studies that could be conducted on this topic.

Existing data indicate that non-problematic video game use is not typically driven by a need to escape real-life concerns or unmet social needs [98]. While some studies link digital gaming with increased severity of anxiety, depression, aggression, and impaired family and personal relationships [8,91], recent systematic reviews suggest that certain types of video games can offer neurostructural and neurofunctional benefits [99] and reduce stress, anxiety, depression, and loneliness, particularly during the pandemic [100]. Therefore, developing a predictive model for general video game time may be less valuable than identifying factors that contribute to problematic gaming behaviors. However, our study observed low average gaming time among girls (0.4 h/day during school days and 0.662 h/day outside school), which limits the ability to draw firm conclusions about problematic use.

Anxiety-related traits such as separation anxiety, social anxiety, and harm avoidance significantly influenced gaming time compared to other variables. The literature supports the notion that avoidance of real-life problems and compensation for deficient socialization are strong predictors of problematic gaming [101]. For girls with ADHD who experience significant social anxiety, video games may provide feelings of competence and relatedness. The customization options in many games allow these girls to create a digital identity, potentially alleviating the burden of real-life social evaluation [102]. However, socially anxious individuals often transfer their dysfunctional behaviors into the virtual world, maintaining distance from in-game characters [103] and avoiding challenging tasks to escape evaluation [104]. Additionally, ADHD exacerbates social anxiety, accelerates its onset, and is associated with more psychiatric comorbidities [105].

### 4.4. Other Digital Activities

The low predictive performance of our models for streaming platforms, YouTube, and television usage suggests that these activities are generally driven by rational motives. Some authors have even introduced the concept of “moral panic”, which posits that there may be an exaggerated concern over digital media’s impact on mental health rather than real associations with various mental health disorders in adolescents [15]. Given our results, it is not excluded that this may indeed apply to some of the digital activities, such as those for which predictive models have shown extremely poor predictive performance, but additional studies are needed to replicate these results.

### 4.5. Strengths of the Study

Our study has several notable strengths. Firstly, it highlights the potential involvement of factors minimally discussed in the literature, such as family disputes, separation anxiety, and avoidance tendencies. We also adopted a multifaceted approach to anxiety disorders, which allowed for greater specificity in identifying associations between adolescents’ digital activities and their mental functioning. Furthermore, our study is among the first to utilize robust machine learning techniques, such as Random Forest regression, in this context. This approach aligns with recommendations from other researchers who have noted the underutilization of these statistical procedures in mental health research despite their higher efficiency compared to other regression types [106].

### 4.6. Limitations

However, our study has several limitations. Although we tried to combat variability in digital activities by treating Monday to Friday and weekend patterns separately, digital activity patterns can vary daily, which cannot be accurately reflected by tools that request the average time spent on a typical day. It is also challenging to quantify the time spent on each activity due to high rates of switching between them (e.g., playing a game on a smartphone and receiving a social media notification). Additionally, multitasking is prevalent, with many adolescents engaging in multiple digital activities simultaneously (e.g., using social media while watching a movie), complicating accurate reporting of time allocated to each specific activity. Despite some attempts to develop tools to effectively quantify multitasking, including among adolescents [107], or to quantify screen time by Ecological Momentary Assessment [108], so far, there is no consensus regarding the optimal instrument. Furthermore, we included patients who reported extremely high screen time (up to 19.2 h/day) without certainty whether these reflect “vamping” phenomena [109] or overestimations, although we have tried to prevent bias by asking additional confirmatory questions and by comparing the reporting of the two sources. On the other hand, it is not excluded that some patients may have underestimated their time spent on digital media or that our results are partly influenced by self-selection bias. In addition, our study omits other factors potentially implicated in dictating digital media consumption, such as cultural, socioeconomic, parenting factors, or inter-individual variability in access to digital media. Lastly, our results may be influenced by specific sample characteristics, such as the inclusion of institutionalized patients who may have stricter device usage rules or patients with autism spectrum disorder, where certain reports, like time spent with friends, may lose relevance.

### 4.7. Future Research Directions

Future studies should focus on a single digital activity, targeting more specifically the variables identified as most predictive, alongside potentially relevant factors omitted in the current study. One primary category is parental influences. Although we highlighted the significant contribution of family disputes and aggression to social media use among girls with ADHD, a wide range of additional factors can be considered. This includes the importance of the time parents themselves spend on digital media [40,110] and various parental monitoring practices, which, if inconsistent, can have paradoxical “boomerang” effects [40,111].

Digital media has led to the definition of new constructs involved in the pathogenesis of anxiety disorders, grouped under the umbrella of “digital stress”. These include elements such as approval anxiety, availability stress, FOMO (fear of missing out), connection overload, and online vigilance [112], which are thought to intervene in the relationship between digital media use and psychosocial outcomes [113]. According to some recent studies, digital stress seems to arise particularly from social media, online learning platforms, games, and video streaming [114]. Additionally, the literature discusses “nomophobia”, a situational phobia manifested by separation from a smartphone [115] which, according to recent studies, appears to lead to at least moderate impairment for more than half of adolescents, particularly as a result of social media use, watching videos and surfing the internet, especially among females [116]. Proponents of ecological systems theory emphasize the importance of including factors that may seem very distal, such as community cultural values, the current state of society, and technological progress [55].

Moreover, while screen time was segmented by adolescents’ main digital activities, further probing can provide deeper insights by dividing each of these activities into more components to more accurately reflect what happens during use. For example, a unitary approach to video games can be misleading due to considerable differences dictated by different types of games (MMORPG, FPS, etc.), both in terms of motives for involvement and their consequences on psyche and behavior [101]. Additionally, different uses of social media lead to different outcomes, such as chatting correlating positively with internalization symptoms, while online sociability correlates negatively with such problems, especially in boys [65].

## 5. Conclusions

This study employed the Random Forest technique, a highly effective regression method, to identify key factors predicting the digital habits of adolescents with ADHD, differentiating between school days and weekends/vacations. Our findings indicate that predictive models were notably successful for social media use and, to a lesser extent, for video game use among girls.

Girls with ADHD were found to spend significantly more time on social media, a trend potentially linked to their focus on self-presentation. In contrast, boys distributed their digital activities more evenly across various platforms. Additionally, social media usage increased with age for both genders. Notably, family disputes and aggression emerged as primary predictors of social media use among girls, suggesting that these dynamics may drive them to seek social support online. Furthermore, girls with ADHD may utilize social media to manage somatic symptoms of anxiety or as a coping mechanism in response to avoidant tendencies that limit engagement in other activities. Among ADHD traits, impulsivity was identified as a significant factor influencing social media use during school days for girls, while inattention was more predictive during weekends and holidays.

For boys with ADHD, cyberbullying was a critical predictor of extended social media use, indicating distinct coping strategies between genders; boys are more likely to engage in prolonged online activity in response to aggression. Among the anxiety components examined, only separation anxiety was a predictor for boys’ social media use, with higher severity associated with reduced engagement. Interestingly, treatment with methylphenidate was found to predict a reduction in time spent on social media, independent of the severity of ADHD symptoms. Contrary to our expectations, social anxiety did not significantly predict social media use for either gender.

In terms of video gaming, our findings suggest that this activity is generally normative among adolescents with ADHD and is less influenced by factors such as anxiety, inattention, hyperactivity, family dynamics, or psychosocial issues. When gaming does become problematic, separation anxiety and social phobia were identified as key predictive components, particularly for girls. This supports the hypothesis that girls with ADHD, particularly those exhibiting severe separation anxiety, may engage more with video games to cultivate a sense of competence, relatedness, and virtual identity, thus alleviating the pressures of social evaluation.

## Figures and Tables

**Table 1:** Distribution of diagnoses among study participants.

Diagnosis	Number of Diagnosed Patients
Depressive disorder	43
Generalized anxiety disorder	32
Social phobia	28
Autism spectrum disorder	14
Conduct disorder	8
Obsessive-compulsive disorder	8
Specific phobia	5
Panic disorder	5
Tic disorder	3
Bipolar disorder	3
Anorexia	3

**Table 2:** Means (and standard deviations) of the daily time allocated by adolescents to various non-digital activities.

Non-Digital Activity	Adolescents Reports (h/day)			Parents Reports (h/day)		
	F	M	Total	F	M	Total
Outdoor activities *						
*Weekdays*	1.013 (±1.231)	1.583 (±1.201)	1.278 (±1.244)	1.183 (±1.452)	1.378 (±1.197)	1.273 (±1.337)
*Weekends and holidays*	1.995 (±1.892)	2.739 (±1.701)	2.340 (±1.835)	2.172 (±1.888)	2.256 (±1.684)	2.211 (±1.788)
Activities with friends						
Weekdays *	1.703 (±2.191)	0.884 (±1.315)	1.323 (±1.874)	1.155 (±1.195)	0.967 (±1.392)	1.068 (±1.286)
*Weekends and holidays*	2.621 (±2.675)	1.944 (±2.146)	2.307 (±2.455)	2.092 (±1.786)	1.744 (±1.839)	1.931 (±1.810)
Doing homework						
*Weekdays*	1.313 (±1.238)	1.661 (±1.046)	1.474 (±1.160)	1.457 (±0.981)	1.809 (±1.137)	1.620 (±1.065)
*Weekends and holidays*	1.205 (±1.441)	1.210 (±1.213)	1.207 (±1.333)	1.336 (±1.251)	1.399 (±1.149)	1.365 (±1.199)
**TOTAL NON-DIGITAL ACTIVITIES**						
*Weekdays*	4.029 (±3.007)	4.129 (±2.149)	4.075 (±2.631)	3.794 (±2.284)	4.153 (±2.319)	3.961 (±2.295)
*Weekends and holidays*	5.821 (±4.158)	5.893 (±3.388)	5.855 (±3.801)	5.600 (±3.153)	5.399 (±3.172)	5.507 (±3.147)

Note. * designates a statistically significant result (*p* < 0.05).

**Table 3:** Average time allocated by adolescents to different types of digital activities (in hours/day).

Digital Activity	Adolescents Reports (h/day)			Parents Reports (h/day)		
	F	M	Total	F	M	Total
Using streaming services						
*Weekdays*	0.832 (±0.953); range 0–3.5	0.889 (±1.279); range 0–6	0.858 (±1.110); range 0–6	0.846 (±1.251); range 0–7	0.711 (±0.827); range 0–4	0.783 (±1.072); range 0–7
*Weekends and holidays*	1.144 (±1.296); range 0–5	1.111 (±1.438); range 0–6	1.129 (±1.356); range 0–6	1.413 (±1.731); range 0–8	0.867 (±1.000); range 0–4	1.160 (±1.457); range 0–8
Using social media *						
*Weekdays*	4.014 (±3.257); range 0–16	1.333 (1.438); range 0–6	2.771 (±2.897); range 0–16	3.168 (±2.710); range 0–14	1.155 (±1.453); range 0–6	2.234 (±2.427); range 0–14
*Weekends and holidays*	4.731 (±3.465); range 0–16	1.618 (±1.686); range 0–6	3.287 (±3.180); range 0–16	3.731 (±3.147); range 0–16	1.278 (±1.439); range 0–6	2.593 (±2.779); range 0–16
Using YouTube and similar platforms						
*Weekdays*	1.450 (±1.702); range 0–8	1.557 (±1.221); range 0–6	1.499 (±1.492); range 0–8	1.605 (1.407); range 0–8	1.305 (±1.030); range 0–4	1.465 (±1.249); range 0–8
*Weekends and holidays*	1.612 (±1.838); range 0–8.5	1.838 (±1.429); range 0–5	1.716 (±1.656); range 0–8.5	1.884 (±1.693); range 0–9	1.578 (±1.381); range 0–6	1.742 (±1.556); range 0–9
Playing video games *						
*Weekdays*	0.397 (±0.831); range 0–4	1.411 (±1.267); range 0–6	0.868 (±1.167); range 0–6	0.476 (±0.728); range 0–3	1.372 (±1.343); range 0–6	0.892 (±1.144); range 0–6
*Weekends and holidays*	0.662 (±1.217); range 0–6	1.989 (±1.388); range 0–4	1.278 (±1.453); range 0–6	0.520 (±0.832); range 0–3	1.533 (±1.358); range 0–6	0.990 (±1.213); range 0–6
Watching TV						
*Weekdays*	0.538 (±1.125); range 0–6	0.758 (±0.820); range 0–3	0.640 (±0.997); range 0–6	0.567 (±0.934); range 0–4	0.794 (±0.754); range 0–3	0.673 (±0.859); range 0–4
*Weekends and holidays*	0.696 (±1.506); range 0–9	0.960 (±1.078); range 0–5	0.819 (±1.324); range 0–9	0.711 (±1.185); range 0–5)	1.064 (±1.019); range 0–4	0.875 (±1.120); range 0–5
**TOTAL DIGITAL ACTIVITIES**						
*Weekdays*	7.232 (±4.137); range 0.5–18	5.948 (±3.960); range 0.5–19.2	6.636 (±4.086); range 0.5–19.2	6.663 (±3.906); range 0.95–19	5.337 (±3.475); range 0.5–19	6.047 (±3.752); range 0.5–19
*Weekends and holidays*	8.738 (±4.713); range 0.5–18.5	7.462 (±3.720); range 0–18	8.146 (±4.307); range 0–18.5	8.259 (±4.576); range 1–22	6.320 (±3.637); range 0–19.5	7.360 (±4.258); range 0–22

Note. * designates a statistically significant result (*p* < 0.05).

**Table 4:** Additional information regarding the included sample.

Variable	Yes	No
Practising a sport	55	42
*1 day/week: 16*		
*2–3 days/week: 25*		
*4–5 days/week: 10*		
*6–7 days/week: 4*		
Enforcing a parental screen time schedule	48	49
*<1 h/day: 9*		
*1–2 h/day: 19*		
*2–3 h/day: 16*		
*>3 h/day: 4*		
Less than 7 h of sleep per night	21	76
More than 9 h of sleep per night	83	14
Biparental status	60	37
Family disputes	34	63
Aggression in the family	11	86
Bullying at school	38	59
Cyberbullying	16	81

**Table 5:** Mean raw scores reported on the MASC and ADHD-RS IV.

Scales and Subscales	Score (Mean ± SD)
MASC	
*Physical Symptoms*	15.330 (±5.184)
*Social Anxiety*	13.381 (±7.998)
*Harm Avoidance*	13.763 (±5.184)
*Separation Anxiety*	8.598 (±4.729)
***TOTAL***	**51.072 (±20.830), range 6–90**
ADHD-RS IV	
*Hyperactivity/Impulsivity*	8.021 (±5.374)
*Inattention*	12.278 (±6.191)
***TOTAL***	**20.299 (±10.729), range 2–52**

## Data Availability

Data available on request due to privacy restrictions.

## Supplementary Materials

The following supporting information can be downloaded at https://www.mdpi.com/article/10.3390/jcm13237461/s1, Table S1: Selected hyperparameters; Table S2: Hyperparameter grids with top 10 predictive models for each variable.
