# Supplementary material for: Analysing Digital Engagement Patterns: A Machine Learning Investigation into Social Anxiety Among Adolescents with ADHD
Source: J Clin Med. 2024 Dec 7;13(23):7461. doi: 10.3390/jcm13237461 (PMC11642364; doi:10.3390/jcm13237461)
Supplement: Supplementary file 1 [file jcm-13-07461-s001.zip › Supplementary Table S1_Selected_hyperparameters.pdf]

Supplementary Table S1. Selected hyperparameters.

Dependent variable	mtry	min.node.size	sample.fraction
SocialMediaWk	3	3	0.80
SocialMediaWE	5	5	0.80
Girls_SocialMediaWk	3	3	0.80
Girls_SocialMediaWE	3	5	0.63
Boys_SocialMediaWk	8	3	0.63
Boys_SocialMediaWE	8	5	0.80
VideoGamesWk	8	10	0.80
VideoGamesWE	8	1	0.63
Girls_VideoGamesWk	3	1	0.80
Girls_VideoGamesWE	3	5	0.80
Boys_VideoGamesWk	1	10	0.80
Boys_VideoGamesWE	1	5	0.80
StreamingWk	1	10	0.80
StreamingWE	5	5	0.80
YoutubeWk	1	1	0.80
YoutubeWE	1	3	0.63
TVWk	1	10	0.50
TVWE	1	10	0.50
