# Supplementary material for: Analysing Digital Engagement Patterns: A Machine Learning Investigation into Social Anxiety Among Adolescents with ADHD
Source: J Clin Med. 2024 Dec 7;13(23):7461. doi: 10.3390/jcm13237461 (PMC11642364; doi:10.3390/jcm13237461)
Supplement: Supplementary file 1 [file jcm-13-07461-s001.zip › Supplementary Table S2_Hyperparameter_grids.pdf]

Supplementary Table S2. Hyperparameter grids with top 10 predictive models for each variable.

SocialMediaWk						
**no**	**mtry**	**min.node.size**	**replace**	**sample.fraction**	**rmse**	**perc_gain**
1	3	5	FALSE	0.80	2.388539	2.50142968
2	3	1	FALSE	0.63	2.392351	2.34582948
3	3	3	TRUE	0.80	2.399323	2.06123967
4	3	3	FALSE	0.63	2.400543	2.01145699
5	3	1	TRUE	0.80	2.402164	1.94528009
6	3	3	FALSE	0.80	2.404656	1.84353538
7	3	5	FALSE	0.63	2.406860	1.75358815
8	3	1	FALSE	0.80	2.406887	1.75250028
9	3	10	FALSE	0.80	2.410004	1.62522743
10	5	1	TRUE	0.80	2.411484	1.56481568
**SocialMediaWE**						
**no**	**mtry**	**min.node.size**	**replace**	**sample.fraction**	**rmse**	**perc_gain**
1	5	3	FALSE	0.80	2.461531	1.05404620
2	5	5	FALSE	0.80	2.471635	0.64789613
3	6	5	FALSE	0.80	2.475216	0.50394584
4	5	1	FALSE	0.80	2.477406	0.41590276
5	6	3	FALSE	0.80	2.478190	0.38441975
6	8	10	FALSE	0.80	2.479249	0.34184203
7	6	10	FALSE	0.80	2.480742	0.28182784
8	8	5	FALSE	0.63	2.480789	0.27991900
9	6	1	FALSE	0.80	2.480980	0.27226322
10	5	10	FALSE	0.80	2.481502	0.25128688
**Girls_SocialMediaWk**						
**no**	**mtry**	**min.node.size**	**replace**	**sample.fraction**	**rmse**	**perc_gain**
1	3	10	FALSE	0.80	2.864791	2.19124169
2	8	3	FALSE	0.80	2.875956	1.81005590
3	3	1	FALSE	0.80	2.876378	1.79565494
4	3	5	FALSE	0.80	2.878750	1.71466460
5	3	3	FALSE	0.80	2.879065	1.70391459
6	5	5	FALSE	0.50	2.882443	1.58855675
7	6	5	FALSE	0.50	2.884180	1.52926157
8	3	1	FALSE	0.50	2.885217	1.49385934
9	3	5	TRUE	0.80	2.888332	1.38750791
10	5	3	FALSE	0.50	2.889115	1.36077433
**Girls_SocialMediaWE**						
**no**	**mtry**	**min.node.size**	**replace**	**sample.fraction**	**rmse**	**perc_gain**
1	3	10	FALSE	0.80	2.871232	2.07107021
2	3	5	FALSE	0.80	2.880041	1.77065145
3	5	5	FALSE	0.63	2.883112	1.66589280
4	5	5	TRUE	0.63	2.894101	1.29108218
5	3	5	FALSE	0.63	2.894543	1.27600394
6	3	1	FALSE	0.50	2.896053	1.22451297
7	3	3	FALSE	0.63	2.897133	1.18768277

8	3	1	TRUE	0.63	2.898877	1.12819409
9	3	1	FALSE	0.63	2.899876	1.09411217
10	5	3	TRUE	0.63	2.899927	1.09238492

Boys_SocialMediaWk

no	mtry	min.node.size	replace	sample.fraction	rmse	perc_gain
1	8	1	TRUE	0.80	1.301014	0.8446863732
2	8	1	TRUE	0.63	1.302989	0.6941811796
3	5	3	TRUE	0.63	1.303765	0.6349972058
4	8	3	TRUE	0.80	1.304506	0.5785498705
5	8	3	TRUE	0.63	1.304510	0.5782329599
6	8	5	TRUE	0.63	1.304798	0.5563094471
7	6	3	FALSE	0.63	1.305384	0.5116022120
8	8	10	TRUE	0.63	1.305457	0.5060343815
9	5	3	FALSE	0.50	1.306165	0.4520929270
10	8	5	TRUE	0.50	1.306526	0.4245777116

Boys_SocialMediaWE

no	mtry	min.node.size	replace	sample.fraction	rmse	perc_gain
1	3	5	FALSE	0.80	2.876370	2.02787185
2	3	10	FALSE	0.80	2.876970	2.00744565
3	5	5	FALSE	0.63	2.887054	1.66398303
4	3	3	FALSE	0.63	2.891468	1.51363136
5	5	5	TRUE	0.63	2.895000	1.39333130
6	3	5	FALSE	0.63	2.895556	1.37437861
7	3	1	FALSE	0.80	2.897518	1.30756706
8	3	1	FALSE	0.50	2.902663	1.13230034
9	5	3	TRUE	0.63	2.902673	1.13196287
10	5	5	FALSE	0.80	2.902732	1.12995518

VideoGamesWk

no	mtry	min.node.size	replace	sample.fraction	rmse	perc_gain
1	6	10	FALSE	0.63	1.123334	-0.4440013
2	8	10	FALSE	0.63	1.124828	-0.5775607
3	5	1	FALSE	0.80	1.125062	-0.5985627
4	8	10	FALSE	0.80	1.125192	-0.6101811
5	6	10	FALSE	0.50	1.125995	-0.6819071
6	6	10	FALSE	0.80	1.126231	-0.7030380
7	6	5	FALSE	0.63	1.126525	-0.7293779
8	8	5	TRUE	0.80	1.127059	-0.7770827
9	8	1	FALSE	0.80	1.127685	-0.8330400
10	8	3	TRUE	0.80	1.127727	-0.8368103

VideoGamesWE

no	mtry	min.node.size	replace	sample.fraction	rmse	perc_gain
1	6	1	FALSE	0.50	1.368451	1.446559e+00
2	8	1	FALSE	0.63	1.369357	1.381301e+00
3	8	5	FALSE	0.63	1.369973	1.336918e+00
4	8	3	FALSE	0.63	1.370205	1.320273e+00
5	8	3	TRUE	0.50	1.371916	1.197010e+00
6	8	1	FALSE	0.50	1.372187	1.177529e+00

7	6	1	TRUE	0.80	1.373524	1.081189e+00
8	5	3	TRUE	0.80	1.373578	1.077344e+00
9	8	5	TRUE	0.50	1.374104	1.039450e+00
10	6	3	TRUE	0.80	1.374113	1.038790e+00
			**Girls_VideoGamesWk**			
**no**	**mtry**	**min.node.size**	**replace**	**sample.fraction**	**rmse**	**perc_gain**
1	1	1	FALSE	0.80	0.7938962	4.27533594
2	1	3	FALSE	0.80	0.7974968	3.84119372
3	3	1	TRUE	0.80	0.7995373	3.59515120
4	3	3	TRUE	0.80	0.7996138	3.58593306
5	3	1	FALSE	0.80	0.7996431	3.58239812
6	1	1	FALSE	0.63	0.8001949	3.51586855
7	3	3	FALSE	0.80	0.8016557	3.33972688
8	1	1	TRUE	0.80	0.8017085	3.33336686
9	1	5	FALSE	0.80	0.8025123	3.23643732
10	3	1	TRUE	0.63	0.8027376	3.20928199
			**Girls_VideoGamesWE**			
**no**	**mtry**	**min.node.size**	**replace**	**sample.fraction**	**rmse**	**perc_gain**
1	3	1	FALSE	0.50	1.203527	1.46201681
2	3	3	FALSE	0.80	1.203574	1.45822042
3	5	1	TRUE	0.80	1.204577	1.37605188
4	3	1	TRUE	0.80	1.205252	1.32076307
5	3	3	FALSE	0.63	1.205683	1.28550776
6	5	3	TRUE	0.80	1.205929	1.26538763
7	3	1	FALSE	0.63	1.206067	1.25408724
8	6	3	TRUE	0.80	1.206331	1.23248511
9	1	1	FALSE	0.80	1.206645	1.20674786
10	3	3	TRUE	0.80	1.207062	1.17258156
			**Boys_VideoGamesWk**			
**no**	**mtry**	**min.node.size**	**replace**	**sample.fraction**	**rmse**	**perc_gain**
1	1	10	FALSE	0.80	1.278048	2.86909778
2	3	10	FALSE	0.80	1.280265	2.70060924
3	1	5	FALSE	0.80	1.280661	2.67047674
4	1	3	FALSE	0.80	1.283383	2.46363994
5	3	10	FALSE	0.63	1.284308	2.39331772
6	1	10	FALSE	0.63	1.284557	2.37442675
7	3	5	FALSE	0.80	1.285238	2.32264948
8	3	10	FALSE	0.50	1.286878	2.19800178
9	1	5	FALSE	0.63	1.289279	2.01556327
10	6	10	FALSE	0.50	1.289366	2.00892860
			**Boys_VideoGamesWE**			
**no**	**mtry**	**min.node.size**	**replace**	**sample.fraction**	**rmse**	**perc_gain**
1	1	10	FALSE	0.80	1.409774	4.20592335
2	1	5	FALSE	0.80	1.414133	3.90969520
3	1	10	TRUE	0.50	1.414368	3.89377855
4	1	5	TRUE	0.50	1.415847	3.79326699
5	1	10	TRUE	0.80	1.416062	3.77866575

6	1	5	TRUE	0.80	1.417797	3.66073266
7	1	3	TRUE	0.50	1.418186	3.63432238
8	1	3	TRUE	0.80	1.418662	3.60196778
9	1	10	FALSE	0.50	1.420209	3.49684326
10	1	5	FALSE	0.63	1.421680	3.39691946
**StreamingWk**						
**no**	**mtry**	**min.node.size**	**replace**	**sample.fraction**	**rmse**	**perc_gain**
1	1	10	FALSE	0.80	1.400140	4.25714034
2	1	5	FALSE	0.80	1.408975	3.65299110
3	1	5	TRUE	0.50	1.415469	3.20893712
4	1	10	TRUE	0.50	1.415579	3.20143722
5	1	3	FALSE	0.80	1.415702	3.19297793
6	1	3	TRUE	0.50	1.417624	3.06160446
7	1	10	FALSE	0.63	1.417706	3.05598060
8	1	10	FALSE	0.50	1.417743	3.05344692
9	1	5	FALSE	0.63	1.419324	2.94535355
10	1	5	TRUE	0.80	1.420053	2.89548817
**StreamingWE**						
**no**	**mtry**	**min.node.size**	**replace**	**sample.fraction**	**rmse**	**perc_gain**
1	5	5	TRUE	0.80	1.306729	1.06457545
2	6	5	TRUE	0.80	1.307500	1.00620481
3	5	1	TRUE	0.50	1.307502	1.00601965
4	5	10	TRUE	0.50	1.307517	1.00488858
5	5	5	TRUE	0.63	1.307597	0.99880281
6	5	10	TRUE	0.80	1.307784	0.98466639
7	6	10	TRUE	0.50	1.307972	0.97047047
8	5	3	TRUE	0.50	1.308227	0.95113004
9	6	1	TRUE	0.50	1.308493	0.93096976
10	5	3	TRUE	0.80	1.308634	0.92028170
**YoutubeWk**						
**no**	**mtry**	**min.node.size**	**replace**	**sample.fraction**	**rmse**	**perc_gain**
1	1	1	FALSE	0.50	1.456665	4.576382355
2	1	3	FALSE	0.80	1.462633	4.185413808
3	1	1	FALSE	0.80	1.464421	4.068278871
4	1	3	FALSE	0.50	1.466054	3.961284614
5	1	5	FALSE	0.80	1.466557	3.928371430
6	1	1	TRUE	0.80	1.467911	3.839625289
7	1	1	TRUE	0.50	1.468503	3.800849077
8	1	1	FALSE	0.63	1.469240	3.752602364
9	1	3	TRUE	0.63	1.469912	3.708567303
10	1	3	TRUE	0.80	1.469974	3.704528574
**YouTubeWE**						
**no**	**mtry**	**min.node.size**	**replace**	**sample.fraction**	**rmse**	**perc_gain**
1	1	3	TRUE	0.63	1.650658	6.54371660
2	1	5	TRUE	0.63	1.651292	6.50779263
3	1	1	TRUE	0.50	1.651866	6.47532890
4	1	3	TRUE	0.50	1.652078	6.46331355

5	1	10	TRUE	0.63	1.652736	6.42603854
6	1	1	TRUE	0.63	1.653276	6.39550004
7	1	5	TRUE	0.50	1.655699	6.25829790
8	1	3	FALSE	0.50	1.658189	6.11733768
9	1	10	TRUE	0.50	1.659002	6.07128887
10	1	5	FALSE	0.63	1.659522	6.04186711
**TVWk**						
**no**	**mtry**	**min.node.size**	**replace**	**sample.fraction**	**rmse**	**perc_gain**
1	1	1	FALSE	0.50	1.007676	4.602379170
2	1	10	TRUE	0.63	1.008299	4.543485208
3	1	10	TRUE	0.50	1.008761	4.499747439
4	1	10	FALSE	0.50	1.008853	4.490962936
5	1	10	TRUE	0.80	1.010201	4.363338897
6	1	1	TRUE	0.63	1.011022	4.285648150
7	1	3	FALSE	0.50	1.011105	4.277799930
8	1	5	TRUE	0.63	1.011419	4.248064430
9	1	5	TRUE	0.50	1.012039	4.189330476
10	1	3	TRUE	0.63	1.012195	4.174649662
**TVWE**						
**no**	**mtry**	**min.node.size**	**replace**	**sample.fraction**	**rmse**	**perc_gain**
1	1	10	TRUE	0.50	1.353993	6.95519771
2	1	10	FALSE	0.50	1.356646	6.77290011
3	1	10	FALSE	0.63	1.360541	6.50526734
4	1	5	TRUE	0.50	1.360684	6.49542651
5	1	10	TRUE	0.63	1.360794	6.48787075
6	1	10	TRUE	0.80	1.361180	6.46132181
7	1	10	FALSE	0.80	1.362834	6.34768906
8	1	3	FALSE	0.50	1.364009	6.26696939
9	1	5	FALSE	0.50	1.364189	6.25455984
10	1	1	TRUE	0.63	1.364220	6.25244102
